# How staff characteristics influence residential care facility staff’s attitude toward person-centered care and informal care

**DOI:** 10.1186/s12912-021-00743-8

**Published:** 2021-11-01

**Authors:** Jogé Boumans, Leonieke van Boekel, Nathalie Kools, Aukelien Scheffelaar, Caroline Baan, Katrien Luijkx

**Affiliations:** Tranzo, Tilburg School of Social and Behavioral Sciences, Tilburg University, PO box 90153, 5000 LE Tilburg, the Netherlands

**Keywords:** Staff characteristics, Attitude, Person-centered care, Informal care, Dementia, Residential care facilities, Older adults

## Abstract

**Background:**

Staff members, and their attitudes, are crucial for providing person-centered care in residential care facilities for people with dementia. However, the literature on the attitudes of nursing staff regarding person-centered care is limited. The objective of this study is to explore the association between staff characteristics (age, education level, years of work experience and function, i.e., care or welfare) and staff attitudes toward perceived person-centered care provision and including informal caregivers in the caregiving process in residential care facilities.

**Methods:**

A convenience sample of 68 care staff – nurses and nurse assistants - welfare staff members – activity counselors, hostesses, and living room caretakers - of two residential care facilities filled out a questionnaire. Staff attitudes regarding perceived person-centered care were measured with the Person-centered Care Assessment Tool (P-CAT). Staff attitudes regarding informal care provision were measured with the Attitudes Toward Families Checklist (AFC). Multiple linear regression analysis explored the association between variables age, work experience, education, and function (care or welfare).

**Results:**

A higher age of staff was associated with a more negative attitude toward perceived person-centered care and informal care provision. Welfare staff had a more negative attitude toward the inclusion of informal caregivers than care staff. The perceived person-centered care provision of the care and welfare staff was both positive. Work experience and education were not associated with perceived person-centered care provision or informal care provision.

**Conclusion:**

This study is one of the first to provide insight into the association between staff characteristics and their attitude toward their perceived person-centered care provision and informal care provision. A higher age of both the care and welfare staff was associated with a more negative attitude toward their perceived person-centered care and informal care provision. Welfare staff had a less positive attitude toward informal care provision. Additionally, future studies, also observational studies and interview studies, are necessary to collect evidence on the reasons for negative attitudes of older staff members towards PCC and informal care giving, to be able to adequately target these reasons by implementing interventions that eliminate or reduce these negative attitudes.

## Background

Over the last decades, the focus in dementia care has been increasingly directed toward person-centered care (PCC). PCC refers to a focus on the care and treatment of the person with dementia and their psychological needs [1] rather than the person’s disease [2]. PCC is advocated as critical for good and effective dementia and nursing home care [3–5], showing significant benefits with respect to decreasing behavioral symptoms [6], psychotropic medication use [7], and improved quality of life for people with dementia [8, 9].

Staff members and their attitudes are crucial when providing PCC in residential care facilities for people with dementia because in the care relation, they have to place the person with dementia at the center of the care dynamic rather than placing the emphasis on the condition [2]. However, the literature on the attitudes of nursing staff regarding PCC is limited. Most studies have focused on how to increase PCC delivery [10, 11] and how PCC influences job satisfaction of the staff. Studies have shown that care staff with more positive attitudes regarding PCC are more satisfied with their jobs [12–17].

Several studies show that characteristics of staff members, such as age, education level, and working experience in long-term care for older adults could influence their PCC attitude though the results are mixed. A study reported that staff over the age of 50 had less person-centered attitudes than staff under the age of 40 [18]. Oppositely, to other studies found that age was not associated with attitudes toward residents with dementia [19, 20]. Also, the role of education is researched in several studies. Moreover, previous research focusing on the role of education found that higher education levels were related to more positive attitudes toward residents with dementia [12, 19]. As for research focusing on the effect of work experience on attitudes, results are rather mixed. One study showed that staff members who worked between one and two years had a higher PPC attitude in comparison with staff members having more work experience [12]. This is in contrast with another study which reported that staff with less than 10 years of experience had less person-centered attitudes [18]. Two other studies found that attitudes did not differ by work experience in long-term care [19, 20]. No studies regarding the function of the staff member (i.e., care or welfare) have been located. Though, the function of the staff member might also influence their PCC attitude. For example, for welfare staff it might be easier to focus on the person with dementia and their psychological needs rather than the person’s disease because they are not or less involved in the physical care of the person with dementia.

PCC provision in residential care facilities does not depend on paid staff only. Forming and maintaining good relationships between staff members, clients, and their families is essential to PCC [21]. Families, but also other informal caregivers like friends and neighbors, are familiar with the preferences of the resident [22], and their involvement in care could lead to more PCC for the person with dementia. A few studies have been conducted on staff attitudes toward family members of residents with dementia [23–25], with all indicating that a positive attitude on the part of staff regarding family members is important for the resident’s well-being. Whether the individual staff characteristics influence the attitude of staff regarding the inclusion of informal caregivers is yet unknown because no study has been located that has investigated the correlations between staff characteristics and staff attitude regarding the inclusion of informal caregivers.

There is a need for empirical measures to determine the association between attitudes of staff regarding PCC and the attitudes of staff regarding informal care. A better understanding of unique individual factors that underlie and contribute to attitudes may enable more targeted staff training in the area of PCC and the inclusion of informal caregivers.

Therefore, the objective of this study is to explore the association between staff characteristics (age, education level, years of work experience and function, i.e., care or welfare) and staff attitudes toward perceived person-centered care provision and including informal caregivers in the caregiving process in residential care facilities. Our research questions were as follows:

1. What is the association between staff characteristics (age, education level, years of work experience in long-term care for older adults and function, i.e., care or welfare) and staff attitudes toward PCC provision?
2. What is the association between staff characteristics (age, education level, years of work experience in long-term care for older adults and function, i.e. care or welfare) and staff attitudes toward the inclusion of informal caregivers?

## Methods

### Participants and settings

A cross-sectional convenience sample of a total of 68 staff members (care staff [*n* = 36] and welfare staff [*n* = 29] missing functions [*n* = 3]) of residential aged care staff was recruited within two psychogeriatric care units of residential care facilities (RCFs) located in the south of the Netherlands (RCF A *n* = 45; RCF B *n* = 23). All levels of (care) staff at these facilities were considered eligible for participation. We also intentionally aimed to include staff who provided non-care tasks but assisted residents with daily activities since they are essential in PCC. Non-care staff is hereafter referred to as welfare staff. Staff were invited by their team manager to participate voluntarily and were informed about the process and aim of the study. We did not track non-responses or reasons why staff members did not participate in the study. Participants completed the questionnaire on paper or online.

Both RCFs provide 24-h care for people with dementia and take a person-centered approach to care. RCF A provides care for people with light to moderate dementia or somatic problems. To live in RCF A, an indication for long-term care is needed. A diagnosis of dementia is not necessary. RCF B provides care mostly for people with moderate to severe dementia. People are eligible to reside and receive care in RCF B if they have a diagnosis of dementia and need long-term care. RCF A and B qualify as small-scale living facilities in the area of a larger nursing home [26]. They both provide care for people with dementia in a homelike situation. A maximum of eight residents form a joint household. A common living room is provided, including a kitchen in which all meals are prepared. Facilities such as a restaurant and activity areas are attached to the ward. The physical environment of the two care organizations regarding the use of PCC elements has been mapped to see if the work conditions of staff members with respect to the environment were compatible with providing PCC.

### Measuring instruments

#### Physical environment

The physical environment of both care locations has been systematically mapped by means of the OAZIS-Dementia [27]. The tool was developed to measure the physical environment of long-term care environments in a Dutch setting [26]. With this tool, it is possible to map the extent to which the physical environment meets the conditions for PCC and thus provides an indication of the extent to which the physical environment contributes to the well-being of the residents. The OAZIS-Dementia consists of 72 items, which assess aspects of the environment on a five-point Likert scale, ranging from 1 (not at all) to 5 (completely). The checklist is divided into seven themes: 1) privacy and autonomy, 2) sensory stimulation, 3) view and nature, 4) facilities, 5) orientation and routing, 6) domesticity, and 7) safety. Higher scores indicate a higher probability of the environment meeting the conditions for PCC. Two independent researchers (*n* = 2) observed both care locations and filled out the OAZIS-Dementia for each care location.

#### Staff questionnaires

The Person-Centered Assessment Tool (P-CAT) is a widely used questionnaire with good psychometric properties (i.e., Cronbach’s α = .84), and content and construct validity have been proven to be good [28]. The P-CAT is a questionnaire constructed to evaluate the extent to which staff in residential aged care perceive the care provided as being person-centered. The questionnaire consists of 13 items, rated on a five-point Likert scale ranging from 1 (disagree completely) to 5 (agree completely). In total, 13 items were summed up to generate a total score between 13 and 65, where higher scores indicate a higher perceived PCC attitude. The questionnaire consists of three subscales, namely: 1) the extent to which care is tailored to the individual (Cronbach’s α = 0.81); 2) the extent to which employees experience support from the organization (Cronbach’s α = 0.77); 3. the extent to which the environment is accessible and suitable for people with dementia (Cronbach’s α = 0.31) [28]. The Dutch version of the tool was translated by a research institute and showed appropriate psychometric properties [29]. In our study, the internal consistency of the scale was Cronbach’s α = 0.80. Subscale 1 and subscale 2 had an acceptable internal consistency (both Cronbach’s α = 0.77). Subscale 3 could not be taken together in a separate scale because of an unacceptable internal consistency (Cronbach’s α = 0.48).

The Attitudes Toward Families Checklist (AFC) was used. Reported Cronbach’s α was .74 for the scale [30, 31]. The AFC is designed to assess staff attitudes about family members of residents with dementia [30]. Staff rated the items on a 7-point Likert-type scale (1 = strongly disagree to 7 = strongly agree). An overall sum score of all 16 AFC items was calculated. High scores indicated that the staff had a positive attitude toward family visitation, requests, and caregiving roles for their relatives. The items of the AFC had satisfactory estimates of reliability which were comparable with the internal consistency of previous findings [30]. The AFC consisted of three subscales: 1) calming, reflecting whether family members are seen as having a calming effect or a disruptive effect on residents; 2) partner, reflecting whether family members are seen as equal partners in care or not; and 3) relevant, reflecting whether family members are seen as relevant to residents and staff. For this study, we translated the questionnaire from English to Dutch using a forward and backward translation process using native speakers. Internal consistency of the subscales was not sufficient, and therefore only an overall score was used in the analysis since internal consistency of the overall scale was sufficient (Cronbach’s α = 0.73).

*Staff characteristics:* Gender, age, education level, years of work experience in long-term care for older adults, and function (care or welfare) were assessed. The functions ‘certified nurse’, ‘registered nurse’, ‘nurse assistant’, and other clearly care-related functions mentioned under ‘other’ were classified as care staff (*n* = 36). The functions ‘activity counselor’, ‘hostess’, ‘living room caretaker’, and other welfare-related functions mentioned under ‘other’ were classified as welfare staff (*n* = 28), missing functions (*n* = 3).

### Analyses

SPSS version 24 was used to perform the analyses. Descriptive statistics were used for all staff characteristics to map averages, minimum and maximum values, and standard deviations. Descriptive statistics were used to map scores on the P-CAT and the AFC for the entire sample and separately for care and welfare staff.

The Shapiro-Wilk test was used to check normality. Results indicated that scores on the P-CAT sum score (*W* = 0.95; *p* < 0.05), as well as scores on P-CAT subscale 1 (*W* = 0.96; *p* < 0.05) and the P-CAT subscale 2 (*W* = 0,95; *p* < 0.05) were not normally distributed. Therefore, Mann-Whitney U tests were used to compare sum scores of P-CAT, P-CAT subscale 1 (‘extent to which care is tailored to the individual’) and P-CAT subscale 2 (‘the extent to which staff experience support from the organization’) between care and welfare staff. Other assumptions for Mann-Whitney U test were not violated since there were no outliers and observations were independent. Overall scores on the AFC were normally distributed, and therefore an independent *t*-test was used to compare scores on AFC between care and welfare staff.

Two multiple linear regressions by means of the enter method were used to investigate first whether overall score on P-CAT could be explained by ‘age of staff member’, ‘education level’ ‘number of years that staff member has been working in long-term care for older adults’ and ‘function of staff member (care or welfare)’. Second, multiple linear regression was used to assess whether the overall score on AFC could be explained by ‘age of staff member’, ‘education level’, ‘number of years that staff member has been working in long-term care for older adults’, and ‘function of staff member (care or welfare)’. Assumptions for multiple linear regression were tested for both multiple regression analyses to explain P-CAT and AFC. Multicollinearity was not a problem since VIF values were between 1 and 2 and tolerance was not below 0.1. Durbin-Watson test was 2.122 (P-CAT as the dependent variable) and 1.991 (AFC as the dependent variable), indicating that errors were uncorrelated. The assumption of homoscedasticity of variances, linearity, and non-zero variances were also met. One respondent had an outlier (rather low) score on overall P-CAT score. We decided, however, to maintain this respondent in the analyses since answers seemed realistic.

### Ethics

The study has been approved by the Ethics Review Board of the School of Social and Behavioral Sciences of Tilburg University (EC-2019.06) and by the ethics committee of residential care facility B. Written informed consent was obtained from all subjects involved in the study.

## Results

The results section consists of six parts: 1) the physical environment regarding PCC, 2) demographics and job-related characteristics of the staff members, 3) results of the P-CAT questionnaire, 4) associations between staff characteristics and perceived provision of PCC, 5) results of the AFC questionnaire, 6) associations between staff characteristics and attitude toward informal care provision.

### Comparison of the physical environment regarding PCC

To gain insight into whether the physical environment of the two care organizations differ from each other or have considerable similarities regarding the use of PCC elements, the physical environment of both care locations has been systematically mapped by means of the OAZIS-Dementia. Results of the systematic mapping of the physical environment regarding PCC are shown in Table 1. RCF B scored (slightly) higher on almost all themes than RCF A, with the exception of the view and nature theme. Both RCFs scored relatively high on the themes privacy and autonomy, facilities, domesticity and relatively low on the themes view and nature and orientation and routing, though the physical environment of both RCFs met most of the conditions to realize PCC.

**Table 1:** Results of the observation of the physical environment by means of the OAZIS-Dementia

	Residential care facilities			
	RCF A (*n* = 2)		RCF B (*n* = 2)	
Themas	*M*	*SD*	*M*	*SD*
1. Privacy and Autonomy	4.14	.00	4.86	.00
2. Sensory Stimulation	4.36	.04	4.37	.06
3. View and Nature	3.72	.12	3.23	.32
4. Facilities	4.11	.00	4.72	.08
5. Orientation and Routing	3.64	.10	4.18	.45
6. Domesticity	4.50	.12	4.59	.00
7. Safety	4.00	.00	5.00	.00
Total	4.07	.01	4.42	.11

*Notes: M* = Mean *SD* = Standard deviation

### Demographics and job-related characteristics of the staff members

A total of 68 staff members (care staff [*n* = 36] and welfare staff [*n* = 29], missing functions [*n* = 3]). completed the questionnaire; 45 staff members from RCF A and 23 staff members from RCF B. Three staff members mentioned functions that could not be classified as a care or welfare function and were therefore coded as missing in the dichotomous score for care or welfare function.

The demographics and job-related characteristics of the staff members are shown in Table 2. All respondents had Dutch nationality. Of these respondents, 90.9% were women. The mean age of the respondents was 42.9 (*SD* = 11.9). When comparing care and welfare staff, it was found that care staff had more years of experience in care for older adults (*M* = 14.8; *SD* = 10.7) compared to welfare staff (*M* = 8.83; *SD* = 10). Due to the low response of male respondents, the correlation between gender and staff attitudes could not be studied.

**Table 2:** Demographics and job-related characteristics of the staff members

Variables	Care staff *(N* = 36*)*	Welfare staff (*N* = 29)	Total (*N* = 68)^a^
	*n* (%)	*n* (%)	*n* (%)
Age (in years) (*SD*)^b^	40.4 (12.0)	46.6 (11.0)	42.9 (11.9)
Gender			
Female	33 (90.9%)	25 (88%)	58 (89.6%)
Years of experience in long-term care for older adults *M (SD)*	14.8 (10.7)	8.83 (10.0)	12.6 (SD = 11,1)
Number of hours worked per week *M* (*SD*)	27.8 (5.3)	25.7 (5.2)	26.6 (5.3)
Education level			
Low (no education, elementary and vocational education)	6	7	13
Middle (secondary and average vocational education)	26	20	46
High (high vocational education, high professional education university)	4	2	6

*Notes:* M = Mean. SD = Standard deviation. ^a^ Total (*N* = 68) = *Care staff (N* = 36*)* + Welfare staff (*N* = 29) + missing functions (*N* = 3) ^b T^Age significantly different between care and welfare staff t(61) = − 2.115 *p* < 0.05

#### Results of P-CAT questionnaire

Attitudes toward PCC of both care and welfare staff members are presented in Table 3. The answers of four respondents are missing. Both care and welfare staff were convinced that they provided PCC to a relatively high degree (P-CAT *M* = 3.82; *SD* = 0.46). The difference between the two types of staff members in the extent to which they were convinced that they provided PCC was investigated using Mann-Whitney U tests. Care staff members (*M* = 3.69) did not differ in their perceived provision of PCC compared to welfare staff members (*M* = 3.77), *U* = 467.00, *p* > .05, *r* = −.06.

**Table 3:** Descriptive statistics P-CAT

P-CAT	*N* =	Range (minimum – maximum)	Mean (*SD*)	Median	Care staff mean (*N)*^*d*^	Welfare staff mean (*N)*
OVERALL SCORE	67	3 (2–5)	3.82 (0.46)	3.69	3.89 (36)	3.75 (28)
Items						
SUBSCALE 1 EXTENT OF PERSONALIZING CARE ^a^	67	3 (2–5)	3.98 (0.480)	3.86	4.07 (36)	3.86 (28)
1. We often discuss how to give person-centered care.	66	3 (2–5)	4.00 (0.823)	4.00	4.11 (36)	3.79 (28)
2. We have formal team meetings to discuss residents’ care.	67	4 (1–5)	3.95 (0.697)	4.00	4.04 (36)	3.79 (28)
3. The life history of the residents is formally used in the care plans we use.	67	4 (1–5)	3.54 (0.943)	4.00	3.64 (36)	3.39 (28)
4. The quality of the interaction between staff and residents is more important than getting the tasks done.	67	3 (2–5)	4.07 (0.724)	4.00	4.11 (36)	4.04 (28)
5. We are free to alter work routines based on residents’ preferences.	67	2 (3–5)	4.28 (0.545)	4.00	4.42 (36)	4.14 (28)
6. Residents are offered the opportunity to be involved in individualized everyday activities.	67	2 (3–5)	4.10 (0.606)	4.00	4.17 (36)	4.07 (28)
7. Assessment of residents’ needs is undertaken on a daily basis.	67	3 (2–5)	3.90 (0.800)	4.00	4.03 (36)	3.79 (28)
SUBSCALE 2 AMOUNT OF ORGANIZATIONAL SUPPORT	67	3 (2–5)	3.79 (0. 723)	4.00	3.791 (36)	3.86 (28)
8. I simply do not have the time to provide person-centered care.	67	3 (2–5)	3.85 (0.839)	4.00	3.92 (36)	3.82 (28)
9. The environment feels chaotic.	67	4 (1–4)	3.48 (0.877)	4.00	3.50 (36)	3.54 (28)
10. We have to get the work done before we can worry about a homelike environment.	67	4 (1–5)	3.82 (1.072)	4.00	3.78 (36)	3.93 (28)
11. This organization prevents me from providing person-centered care.	67	4 (1–5)	4.03 (0.953)	4.00	3.97 (36)	4.14 (28)
SUBSCALE 3 DEGREE ENVIRONMENTAL ACCESSIBILITY ^c^						
12. It is hard for residents in this facility to find their way around.	67	3 (2–5)	3.19 (0.892)	3.00	3.25 (36)	3.07 (28)
13. Residents are able to access outside space as they wish.	67	4 (1–5)	3.45 (1.118)	4.00	3.61 (36)	3.29 (28)

^a^ Cronbach’s a for subscale 1 was .77

^b^ Cronbach’s a for subscale 2 was .77

^c^ Cronbach’s a for subscale 3 was .48. Because of low internal consistency, this subscale was not further used in the analyses

^d^ Three staff members mentioned functions that could not be classified as a care or a welfare function

The difference between two types of staff members (care or welfare) in the extent to which they offered personalized care (P-CAT subscale 1) was investigated. Care staff (*M* = 4.00) did not differ in the extent to which they personalized care compared to welfare staff (*M* = 3.86), *U* = 390.00, *p* > .05, *r* = −.19. The difference between care and welfare staff in the extent to which staff experience support from their organization (P-CAT subscale 2) was investigated. It appeared that care staff (*M* = 3.75) did not differ in the extent to which they experience support from their organization compared to welfare staff (*M* = 4.00), *U* = 427.50, *p* > .05, *r* = −.13.

#### Associations between staff characteristics and perceived provision of PCC

Table 4 displays the results of the two multiple linear regression analyses in which we investigated whether the attitude of staff members toward PCC provision (P-CAT) could be explained by the staff member’s age, years of work experience in long-term care for older adults, function (care or welfare), and education level. We found that the age of staff members, number of years working in long-term care for older adults, function (care or welfare), and education level explained 16% of the variance in the attitude of staff toward their conviction about their own PCC provision: *F*(4, 56) = 2.628, *p* < .05, *R*^*2*^ = .158 R^2^Adjusted = .098. Years of work experience in long-term care for older adults, function (care or welfare), and education level were not significant explanation of staff attitudes about their perceived PCC provision. The staff’s age was an explanation of the attitude of staff members about their perceived PCC provision (*β* = −.37, *t*(56) = − 2.16, *p* < .05). This represented a negative effect, which means that a higher age of staff members is associated with a more negative attitude toward their perceived PCC provision.

**Table 4:** Results of multiple linear regression analyses to explain attitude of staff toward PCC (P-CAT) and toward informal caregiving (AFC)

Dependent variable attitudes of staff toward PCC (P-CAT)					
	***B***	***SE B***	***β***	t	***p***
*Variable*					
Constant P-CAT	4.927	0.623		11.391	.000
Function: care or welfare	−0.086	0.135	−0.90	−.632	.530
Age staff member	−0.15	0.007	−0.365	−2.156	.035*
Years working in long-term care for older adults	−0.001	0.007	−0.034	−.206	.838
Education level	−0.081	0.059	−.176	−1.372	.175
Dependent variable attitudes of staff toward informal caregiving (AFC)					
	***B***	***SE B***	***β***	**t**	***p***
*Variable*					
Constant AFC	5.025	0.623		8.074	.000
Function: care or welfare	−.107	0.196	−.079	−.546	.587
Age staff member	−.022	.010	−.385	− 2.245	.029*
Years working in long-term care for older adults	.014	.010	.216	1.312	.195
Education level	.045	.085	.069	.531	.598

*Notes*. B = unstandardized coefficients, *SE* = standard error, β = standardized coefficients

P-CAT: *R*^2^ = 0.158, Adjusted *R*^*2*^ = 0.098 *F*(4, 56) = 2.628.

AFC: *R*^*2*^ = 0.154, Adjusted *R*^*2*^ = 0.093 *F*(4. 55) = 2.505.

#### Results of AFC questionnaire

Attitudes toward informal care provision of both care and welfare staff members are presented in Table 5. For each item of this questionnaire, 4–5 responses were missing. The difference between two types of staff (care or welfare staff) regarding their attitudes toward informal care provision was investigated using an independent *t*-test. Care staff had a significantly more positive attitude toward informal care provision (*M* = 4.57; SE = .11) compared to welfare staff (*M* = 4.21; *SE* = .12), *t*(62) = 2.14, *p* < .05. This represented a small to medium effect, *r* = .26.

**Table 5:** Descriptive statistics AFC

AFC^a^	*N* =	Range (minimum – maximum)	Mean (*SD*)	Care staff mean *(N)*^*b*^	Welfare staff mean *(N)*
Items					
1. Family members make too much noise and disturb other residents with Alzheimer’s.^a^	67	5 (1–6)	3.40 (1.338)	3.47 (36)	3.29 (28)
2. It seems that when families come to the Alzheimer’s unit, the residents get more agitated.^a^	67	5 (2–7)	3.54 (1.295)	3.58 (36)	3.54 (28)
3. Family members should remember that they are visitors at the institution and should strictly follow our rules.	65	6 (1–7)	3.57 (1.912)	3.97 (35)	3.07 (27)
4. The institution’s rules about family member visits should be more strict.^a^	66	5 (2–7)	5.18 (1.435)	5.39 (36)	3.96 (27)
5. When families are with their relatives, they often stay too long.^a^	66	5 (2–7)	5.32 (1.361)	5.49 (35)	5.18 (28)
6. Family members often bring ideas that are helpful about how to care for their relatives.	67	5 (2–7)	4.7 (1.206)	4.75 (36)	4.54 (28)
7. Working with the family is an important part of my work.	67	5 (2–7)	5.85 (0.942)	5.97 (36)	5.61 (28)
8. Family members are good about helping with the care of the residents with Alzheimer’s.	67	5 (2–7)	4.85 (1.258)	5.08 (36)	4.50 (28)
9. Family members should have as much say as possible concerning the care of their relatives.	67	6 (1–7)	4.55 (1.699)	4.97 (36)	3.93 (28)
10. Most family members rarely come to see their relatives with Alzheimer’s.^a^	67	5 (2–7)	4.30 (1.337)	4.42 (36)	4.07 (28)
11. Most family members won’t accept that their relatives with Alzheimer’s are mentally incompetent.^a^	67	6 (1–7)	3.84 (1.298)	3.92 (36)	3.75 (28)
12. Family members have too many requests that make my work more difficult.^a^	67	6 (1–7)	3.91 (1.535)	4.11 (36)	3.54 (28)
13. Most family members know a lot about how to relate to their relatives with Alzheimer’s.	66	6 (1–7)	4.08 (1.328)	4.20 (35)	3.93 (28)
14. When family members are on the Alzheimer’s unit, they seem to not know what to do.^a^	66	5 (2–7)	4.35 (1.330)	4.47 (36)	4.22 (27)
15. Family members understand that we care for a number of residents with Alzheimer’s and cannot always do the things they request.	67	5 (2–7)	4.03 (1.359)	4.03 (36)	4.04 (28)
16. Most residents with Alzheimer’s ignore their families that are with them and don’t seem to care if they are there or not.^a^	67	5 (2–7)	5.22 (1.391)	5.14 (36)	5.29 (28)

^a^ Cronbach’s a for the overall scale was 0.73

^b^ Three staff members mentioned functions that could not be classified as a care or a welfare function

#### Associations between staff characteristics and attitude toward informal care provision

Table 4 shows the results of the multiple linear regression in which we investigated whether the attitude of staff members toward informal care provision (AFC) could be explained by the staff member’s age, years of work experience in long-term care for older adults, function (care or welfare), and education level. We found that the age of the staff member, the number of years working in long-term care for older adults, function (care or welfare) and the education level explained 15% of the variance in the attitude of staff member toward informal care: *F*(4. 55) = 2.505, *p* > .05, *R*^*2*^ = .154, R^2^Adjusted = .093. Function (care or welfare), years of work experience in care for older adults, and education level were not significant explanation of staff attitudes to informal care provision. The staff member’s age was an explanation of the attitude that staff had about informal caregiving (*β* = −.39, *t*(55) = − 2.25, *p* < .05). This represented a negative effect, which means that a higher age of staff members is related to a more negative attitude toward informal caregiving.

## Discussion

This article provides insight into the association between staff characteristics and the attitudes of staff regarding providing PCC and the inclusion of informal caregivers. Staff members with a positive attitude toward PCC and the inclusion of family members in care could lead to more PCC for persons with dementia, which contributes to residents’ well-being.

Findings showed that in a physical environment that met most of the conditions to realize PCC, both care and welfare staff were convinced that they provide PCC to a relatively high degree. Also, staff members held a rather positive attitude regarding informal care provision. When comparing care and welfare staff, we found that care staff held a more positive attitude toward informal care provision than welfare staff. Furthermore, a higher age of staff was related to more negative attitudes about their perceived PCC provision and informal care provision. Years of work experience in long-term care for older adults and education level were not significant explanations of staff attitudes toward their perceived PCC provision or informal care provision. Due to the low response of male respondents, the correlation between gender and staff attitudes was not possible to study.

When comparing the study findings to previous studies, the finding that older staff members take on a less person-centered attitude has been confirmed elsewhere [18]. Still, this finding seems surprising at first sight. It could be reasoned that older staff members have more experience in caring for people with dementia and therefore might have more opportunities to gain knowledge and skills through experience and on-the-job training and therefore have a more positive attitude regarding PCC. An explanation for the opposite finding may be that due to the focus of dementia care being on PCC over the last decades, younger care and welfare staff are increasingly being trained in PCC. In another study, older staff members, relative to younger staff members, showed significantly higher rates of emotional exhaustion and showed a tendency toward higher feelings of depersonalization and lower rates of personal accomplishment [32]. Moreover, younger staff may adopt a more optimistic attitude and suffer less from exhaustion.

Our results about the role of education could not confirm the existing research that care staff with higher levels of education have more positive attitudes towards residents with dementia [12, 19]. Our results regarding the years of work experience in long-term care for older adults do confirm the findings of two other studies which stated that attitudes regarding PCC did not differ by years of work experience in long-term care [19, 20]. This seems odd because it could be reasoned that staff who have worked longer with dementia patients may have gained knowledge education via on-the-job training and through experience, thereby improving their attitudes. A reason could be that recently graduated care staff have received more education about PCC than care staff who graduated longer ago. Though, age and number of years of work experiences do not always have to go hand in hand. For example, research facility A had a lot of lateral entrants who had worked in other sectors before and later in their life changed their career to work in the care sector.

The results of our study did not indicate that the function of the staff member (i.e., care or welfare) had much influence on their perceived PCC attitude. Both types of staff members indicated that they thought that they provided PCC to a high degree. Other elements could be of influence on their attitude regarding people with dementia, like on-the-job training or the physical environment. Both research facilities qualify as small-scale living facilities and a person-centered attitude to care is more often reported by staff working in small-scale living facilities [12, 27, 33]. This could mean that because all staff was working in a small-scale living facility their function, level of education and years of working experience had less influence on their attitude than their working environment, and might be an explanation of the findings.

With regards to informal care our study indicated that care staff had more positive attitudes toward informal care provision than welfare staff. There is not much research available about these different stances of care staff and welfare staff. In a study [34], among staff of rural nursing homes, a comparison was made between nurses, aides, and activity workers concerning their experience of job strain. The results showed that activity workers felt a particular responsibility to meet residents’ needs for social interaction and meaningful activity and felt extra pressure to meet the multiple and conflicting demands of different groups of residents. Due to this ambivalence, the function of an activity or welfare staff member regarding informal care provision could be more difficult than that of a care staff member. Care staff usually take care after one resident at a time, whereas, activities organized by welfare staff often concern group-focused activities for residents. This means that the demands of several residents should be considered at the same time, including individual wishes from family members, or including informal caregivers could make a complex situation even more complex. This might explain the less positive attitude of welfare staff toward informal care provision.

### Strengths and limitations

This study has some limitations. We are aware that the sample size is rather small. A convenience sample of 68 staff members of only two RCFs from the southern part of the Netherlands was included. Both RCFs qualify as a small-scale living facility in the area of a larger RCF. This limits the generalizability of the study results because small-scale living is only one of the several different contexts in which care for people with dementia can be provided. Due, however, to the same setting of the RCFs, we were able to gain insight into the specific physical environment of the care organizations regarding the use of PCC elements. The demographic characteristics of the current sample were consistent with those of the Dutch nursing sector [35, 36] regarding the characteristics of sex, mean age, and average number of years of work experience, which supports the likeliness of the representativeness of the sample. Also, this study is a perceived result by questionnaire rather than observation of person-centered care for dementia patients by staff. Observational studies retrieve more standardized information collected by an observing researcher instead of subjective staff responds in the self-completed questionnaire.

A strength of this study is that we made use of existing and validated questionnaires. In addition, our study included both care and welfare staff members. Often, studies regarding PCC and informal care include only care staff members, whereas welfare staff members also are involved and important in PCC provision and including informal caregivers.

### Relevance to clinical practice

This study is one of the first to provide insight into the association between staff characteristics and their attitude toward their perceived PCC provision and informal care provision. The results of the study show that the characteristics of staff members (i.e., age and being care or welfare staff) provide an indication of whether staff members are more inclined to provide PCC or include informal caregivers.

Future studies are necessary to collect evidence on the reasons for the negative attitudes of older staff members toward their perceived PCC provision and informal care provision. Future studies are also necessary to collect evidence on if and/or why years of work experience in care for older adults and education level are not explaining staff attitudes to PCC provision and informal care provision. Observational studies and interview studies need also be considered. Observational studies retrieve more standardized information collected by an observing researcher instead of subjective staff responses on the self-completed questionnaire. And interview studies with staff members could gain insight in their perceptions how and why age, years of work experience in care for older adults, function and education level are or are not of influence on the attitude of staff members regarding PCC and informal care. The facility culture of a dementia care facility can evolve over time (37) and administrators of dementia care facilities may be able to adequately target the reasons for the negative attitudes of staff members regarding PCC and informal care by implementing interventions that eliminate or reduce these negative attitudes.

## Conclusion

New insight into the association between staff characteristics and their attitude regarding perceived PCC provision and informal care provision are given. A higher age of both care and welfare staff was associated with a more negative attitude toward their perceived PCC and informal care provision. Welfare staff had a less positive attitude regarding informal care provision. Additionally, future studies are necessary to collect evidence on the reasons for negative attitudes of older staff members towards PCC and informal care giving, to be able to adequately target these reasons by implementing interventions that eliminate or reduce these negative attitudes.

## Data Availability

The datasets generated and/or analysed during the current study are available from the corresponding author on reasonable request. The data that support the findings of this study are available from De Leyhoeve but restrictions apply to the availability of these data, which were used under license for the current study, and so are not publicly available. Data are however available from the authors upon reasonable request and with permission of De Leyhoeve.

## Abbreviations

PCC: person-centered care
RCFs: residential care facilities
P-CAT: Person-Centered Assessment Tool
AFC: Attitudes Toward Families Checklist
